# Corrigendum: *Jejubacter calystegiae* gen. nov., sp. nov., moderately halophilic, a new member of the family *Enterobacteriaceae*, isolated from beach morning glory

**DOI:** 10.71150/jm.2508100

**Published:** 2025-08-13

**Authors:** Lingmin Jiang, Dexin Wang, Jung-Sook Lee, Dae-Hyuk Kim, Jae Cheol Jeong, Cha Young Kim, Suk Weon Kim, Jiyoung Lee

**Affiliations:** 1 Korean Collection for Type Cultures, Biological Resource Center, Korea Research Institute of Bioscience and Biotechnology, Jeongeup 56212, Republic of Korea; 2 Department of Bioactive Materials, Jeonbuk National University, Jeonju 54896, Republic of Korea; 3 Radiation Utilization and Facilities Management Division, Korea Atomic Energy Research Institute, Jeongeup 56212, Republic of Korea

**Corrigendum: Journal of Microbiogy. 2020. 58: 357–366.**

https://doi.org/10.1007/s12275-020-9294-1

The original online version of this article was revised:

This article contained an error in the Acknowledgments section.

## Acknowledgments

### The originally published version read as follows:

We thank Dr. Aharon Oren for his assistance with the nomenclature and etymology. We also thank Dr. Belinda C. Ferrari for providing the full-length sequence of the 16S rRNA gene of *I. australiensis* KCTC 72143T. This work was carried out with support from the KRIBB Research Initiative Program (KGM5281913).

### It should be read as follows:

We thank Dr. Belinda C. Ferrari for providing the full-length sequence of the 16S rRNA gene of *I. australiensis* KCTC 72143T. This work was carried out with support from the KRIBB Research Initiative Program (KGM5281913).

